# Retraction: The long non-coding RNA SNHG5 regulates gefitinib resistance in lung adenocarcinoma cells by targetting miR-377/CASP1 axis

**DOI:** 10.1042/BSR20180400_RET

**Published:** 2026-07-30

**Authors:** ZheXing Wang, LiMing Pan, HaiXiang Yu, Yue Wang

**Affiliations:** 1 Department of Thoracic Surgery, China-Japan Union Hospital of Jilin University, Changchun, Jilin Province 130033, P.R. China; 2 Department of Oncology, The First Hospital of Jilin University, Changchun, Jilin Province 130021, P.R. China

**Keywords:** gefitinib, human lung adenocarcinoma, SNHG5

This article is being retracted from *Bioscience Reports* at the request of the Editor-in-Chief and the Editorial Board. This follows the receipt of a notification from a reader, alerting the Editorial Board to concerns regarding the western blots and Table 1. Specifically, these concerns include:

- The Figure 4D GAPDH panel which seems to appear in part as Figure 4F GAPDH after a rotation transformation
- The Figure 4F GAPDH bands appear highly similar to the following:
  ○ The Figure 5I B-actin bands of Liu et al. 2017 (DOI: 10.2147/OTT.S124595)
  ○ The Figure 4A B-actin bands of Zhang et al. 2017 (DOI: 10.1007/s12038-017-9698-1)
- The first two Figure 4F CASP1 bands appear highly similar to the following:
  ○ The first two Figure 4A SIRT1 bands of Zhang et al. 2017.
  ○ The fifth and sixth Figure 4H DNMT1 bands of Li et al. 2017 (DOI: 10.1002/jbt.21933)
- The Figure 4D GAPDH bands appear highly similar to the first six B-actin bands from Figure 4G of Li et al. 2017 (DOI: 10.1002/jbt.21933)
- Background irregularities have been noted in the Figure 4D CASP1 blot
- In Table 1, the EGFR mutation before-treatment group has a total of 33 patients, whilst the after-treatment group has a total of 36 patients.

The authors were contacted and provided original data for the GAPDH Western blots in Figures 4D and 4F. However, this did not resolve the concerns, and upon review, the Editorial Office is concerned that peer review may have been compromised for this article. The authors did not respond to subsequent contact.

Given the extent of the issues raised, the Editorial Board stands by the decision to retract the article.

